# The Impacts of Essential Gcp/TsaD Protein on Cell Morphology, Virulence Expression, and Antibiotic Susceptibility in *Staphylococcus aureus*

**DOI:** 10.3390/microorganisms13092111

**Published:** 2025-09-10

**Authors:** Haiyong Guo, Ting Lei, Junshu Yang, Lin Han, Yue Wang, Yinduo Ji

**Affiliations:** 1 School of Life Science, Jilin Normal University, Siping 136000, China; 2 Department of Veterinary and Biomedical Sciences, College of Veterinary Medicine, University of Minnesota, Saint Paul, MN 55108, USAyang1181@umn.edu (J.Y.)

**Keywords:** *Staphylococcus aureus*, Gcp/TsaD, tRNA modification, antibiotic susceptibility, RNA-seq

## Abstract

Our previous studies identified the Gcp/TsaD protein as essential for *Staphylococcus aureus* survival and implicated it in tRNA modification. Here, we demonstrate its broader role in bacterial physiology. Through a morphological analysis, RNA sequencing, network-based bioinformatics, and antibiotic susceptibility testing, we show that Gcp/TsaD influences cell morphology, cell wall integrity, transcriptional regulation, virulence, and antibiotic response. Gcp/TsaD depletion caused reduced cell size and increased cell wall thickness, suggesting its roles in cell division and peptidoglycan biosynthesis. The kinetic transcriptomic analysis revealed widespread changes in gene expression, particularly in the translation and amino acid biosynthesis pathways, supporting its function in maintaining translational fidelity via tRNA modification. Its depletion also upregulated the genes involved in cell envelope biosynthesis, including capsule formation, enhancing resistance to antimicrobial peptides, while downregulating the key virulence genes, indicating a role in pathogenicity. Functionally, the Gcp/TsaD-deficient cells were more susceptible to fosfomycin, reinforcing its importance in cell wall integrity. Together, these findings highlight the multifaceted contribution of Gcp/TsaD to *S. aureus* physiology and underscore its potential as a therapeutic target, particularly against antibiotic-resistant strains.

## 1. Introduction

*Staphylococcus aureus* is a formidable pathogen capable of causing severe infections in both humans and animals. The increasing prevalence of multidrug-resistant strains, particularly methicillin-resistant *S. aureus* (MRSA), in clinical settings poses a significant public health concern [1,2]. Consequently, gaining a deeper understanding of MRSA’s physiology is critical for developing alternative strategies to combat antibiotic-resistant infections.

Among the potential targets for novel antibacterial agents is the staphylococcal Gcp protein. Gcp and its homologs are essential for the viability of several bacterial species, including *S. aureus* [3,4], *Streptococcus pneumoniae* [3], *Escherichia coli* [5], *Bacillus subtilis* [6,7], *Francisella novicida* [8], *Pseudomonas aeruginosa* [9], and *Mycoplasma genitalium* [10]. These homologs possess diverse functions. For example, the Gcp of *Mannheimia haemolytica* exhibits glycoprotease activity, specifically cleaving O-sialoglycosylated proteins [11]. As members of the ASKHA (acetate and sugar kinases, HSP70, and actin) superfamily, Gcp homologs, such as *Pyrococcus abyssi* Pa-Kae1, can bind iron and ATP and demonstrate DNA-binding and apurinic endonuclease activity [12]. In yeast, the Gcp homolog Kae1 is a core component of the KEOPS/EKC complex, which plays a role in transcription and chromatin regulation [13,14].

Our previous studies have demonstrated that Gcp, also known as TsaD, is indispensable for *S. aureus* viability under in vitro conditions and is involved in regulating bacterial autolysis [4] and the N^6^-threonyl carbamoyl adenosine (t^6^A) modification of tRNA [15]. Furthermore, our proteomic studies have revealed that Gcp modulates the biosynthesis of branched-chain amino acids (BCAAs), which are vital for bacterial growth and metabolism [15]. In *B. subtilis*, BCAA biosynthesis is orchestrated by the *ilvBHC*-*leuABCD* (*ilv*-*leu*) operon and related genes, such as *ilvA*, *ilvD*, *ybgE*, and *ywaA* [16,17,18]. *S. aureus* harbors a homologous gene cluster, *ilvDBHC*-*leuABCD*-*ilvA*, with *ilvE* serving a role analogous to *ybgE* and *ywaA* in *B. subtilis* [15]. Gcp depletion was shown to enhance the production of key enzymes in this pathway, as revealed by a proteomic analysis, and to suppress *ilv*-*leu* operon transcription, based on qPCR, promoter–lux fusions, and gel-shift assays [15]. Notably, Gcp depletion also upregulated CcpA, a known activator of *ilv*-*leu* expression, without affecting the CodY levels [15].

BCAA metabolism plays a critical role in bacterial physiology, as these hydrophobic amino acids are essential for membrane protein structure and function [19,20]. Their intermediates, branched-chain α-keto acids, contribute to membrane biosynthesis via branched-chain fatty acids [21,22], and serve as precursors for cofactors such as pantothenate and coenzyme A [23,24]. To elucidate whether Gcp’s essentiality is due to its repression of the ILV biosynthesis pathway, we deleted the *ilv*-*leu* operon. Surprisingly, this did not alter Gcp’s essential role, suggesting that its function extends beyond ILV regulation [15]. In *E. coli*, Gcp homologs (TsaD) participate in the synthesis of t^6^A, a conserved tRNA modification essential for translation fidelity [25,26]. Our research revealed that Gcp is similarly required for t^6^A biosynthesis in *S. aureus* [15], and its interaction with YeaZ (TsaB) is crucial for bacterial viability [27,28]. Taken together, these findings indicate that Gcp/TsaD is multifunctional in bacteria.

In this study, we further investigated the role of Gcp/TsaD in *S. aureus* by assessing its effects on cell morphology using scanning and transmission electron microscopy. We also performed kinetic transcriptomic analyses via RNA sequencing to examine its global transcriptional impact. Together, these approaches provided a comprehensive view of the multifaceted role of Gcp/TsaD in staphylococcal physiology and highlight potential avenues for therapeutic intervention.

## 2. Materials and Methods

### 2.1. Bacterial Strains, Plasmids, Antibiotics, and Growth Conditions

*S. aureus* WCUH29, a methicillin-resistant clinical isolate (MRSA), was used in this study [15]. The control strain JW29011 (WCUH29 attB::pLH1) and the *gcp*/*tsaD* conditional expression mutant JW290111 (WCUH29 Δ*gcp attB*::*Pspac-gcp*, carrying plasmid pYH4-lacI) were cultured in Tryptic Soy Broth (TSB) at 37 °C with shaking at 225 rpm [15]. The antibiotics were purchased from Sigma-Aldrich (St. Louis, MO, USA). Where applicable, the cultures were supplemented with 5 µg/mL erythromycin, 2.5 µg/mL tetracycline, and 100 µM isopropyl β-D-1-thiogalactopyranoside (IPTG, Sigma-Aldrich) to induce *gcp*/*tsaD* expression.

### 2.2. Scanning Electron Microscopy (SEM)

Overnight cultures of JW290111 were diluted 1:100 in fresh TSB with or without 100 µM IPTG and grown to the mid-log phase (OD600 = ~0.5). The cells were fixed in 2.5% glutaraldehyde in 0.1 M sodium cacodylate buffer (Electron Microscopy Sciences, EMS, Hatfield, PA, USA) overnight at 4 °C, washed, and post-fixed in 1% osmium tetroxide (EMS) in the same buffer. After washing, the samples were dehydrated through a graded ethanol series (25 to 100%), treated with hexamethyldisilazane (HMDS; EMS), air-dried on coverslips, and mounted on SEM stubs. The samples were sputter-coated with platinum (Ted Pella Inc., Redding, CA, USA) and visualized using a Hitachi S3500N scanning electron microscope (Hitachi, Tokyo, Japan). The images were acquired using Quartz PCI software Version 8 (Quartz Imaging Corp., Vancouver, BC, Canada), and the cell surface areas were quantified using iTEM software the 2013 version (Olympus SIS, Münster, Germany).

### 2.3. Transmission Electron Microscopy (TEM)

The bacterial cultures were processed as described for the SEM until the dehydration step, which was performed using an acetone gradient (25 to 100%). The samples were infiltrated with 2:1 acetone–Embed 812 resin (EMS) for 1 h, subsequently transferred to a 1:2 acetone–Embed 812 resin mixture for 1 h, and infiltrated with 100% resin. The resin-embedded samples were polymerized overnight at 58 °C in gelatin capsules. Ultrathin sections (60–70 nm) were prepared using a Leica UC6 ultramicrotome (Deerfield, IL, USA) and mounted on 200-mesh copper grids (EMS). The sections were stained with 5% uranyl acetate and Sato lead citrate, then examined with a JEOL 1200 EX II transmission electron microscope (Peabody, MA, USA) ggplot2. The images were captured using a Veleta 2K × 2K camera (Lakewood, CO, USA) and iTEM software, and measurements of cell area, perimeter, and peptidoglycan thickness were performed using the same software.

### 2.4. RNA Isolation and Purification

Overnight cultures were diluted 1:100 in fresh TSB, with or without 100 µM IPTG, and harvested at OD600 ≈ 0.2 (early), 0.5 (mid-log), and 1.0 (early stationary phase). The total RNA was isolated using either an SV Total RNA Isolation System (Promega, Madison, WI, USA) or a RiboPure™-Bacteria Kit (Thermo Fisher Scientific, Waltham, MA, USA). The genomic DNA was removed with two rounds of TURBO DNase treatment (Ambion, Austin, TX, USA), and the RNA concentration was measured at 260 nm.

### 2.5. RNA Sequencing (RNA-Seq) and Data Analysis

#### 2.5.1. RNA Sequencing

The total RNA from three growth phases was purified from three biological replicates. The ribosomal RNA was depleted using a Ribo-off rRNA Depletion Kit (Bacteria), followed by cDNA synthesis and library preparation with a VAHTS™ Stranded mRNA-seq Library Prep Kit for Illumina^®^ (San Diego, CA, USA). The sequencing was performed on the Illumina platform [29].

#### 2.5.2. Differential Gene Expression Analysis

Differential expression was analyzed using DESeq [30], with significance thresholds set at q-value ≤ 0.05 and |log_2_ fold change| ≥ 1.

#### 2.5.3. Functional Enrichment Analysis

The differentially expressed genes were subjected to a KEGG and Gene Ontology (GO) Biological Process (BP) enrichment analysis using the R language cluster Profiler. Fisher’s exact test was used to evaluate the statistical significance, and visualization was performed using ggplot2.

#### 2.5.4. Protein–Protein Interaction (PPI) Network Analysis

The PPI networks were constructed using the STRING 11.5 database, referencing *S. aureus* NCTC 8325. A confidence score threshold of ≥0.4 was applied. The network diagrams were generated using Cytoscape v3.6.1.

### 2.6. Semi-Quantitative Real-Time RT-PCR (qPCR)

To validate RNA-seq results, selected genes were analyzed by qPCR [29]. Cultures were grown to mid-log phase (OD600 = ~0.5), and RNA was isolated as described. cDNA synthesis was carried out with SuperScript III (Invitrogen, Waltham, MA, USA) and random primers. Reactions were run in duplicate using VeriQuest SYBR Green Master Mix (Affymetrix, Santa Clara, CA, USA) on Stratagene Mx3000P system (Stratagene, La Jolla, CA, USA). Primers (Table 1) were designed to yield 100–200 bp amplicons. Relative expression was calculated using ΔΔCt method, with 16S rRNA as internal control.

### 2.7. Minimum Inhibitory Concentration (MIC) Assays

*S. aureus* strains were grown overnight in TSB, diluted to ~10^5^ CFU/mL in Mueller–Hinton broth (MHB), and exposed to serial dilutions of test compounds in 96-well microtiter plates as described in [31]. MICs were defined as lowest concentration that inhibited visible growth after 18 h incubation at 37 °C.

## 3. Results

### 3.1. Impact of Gcp/TsaD Deletion on Bacterial Cell Morphologies

**Figure 1:**
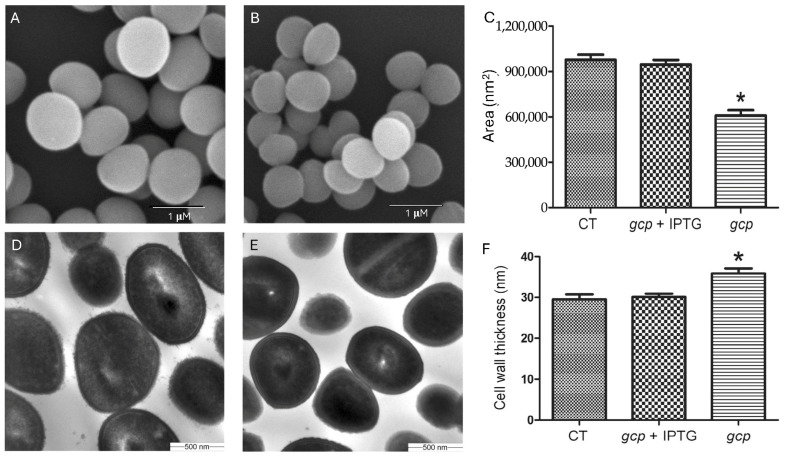
Scanning and transmission electron micrographs of *S. aureus* with Gcp/TsaD depletion. The SEM is of the gcp/tsaD conditional mutant JW290111 grown in TSB with (**A**) and without (**B**) 100 µM IPTG. The scale bar in the SEM represents 1 µm. The bacterial cell size is measured as the area of cells under the SEM (**C**). The cell size is measured for the parental control JW290011 (CT) and the *gcp*/*tsaD* conditional mutant JW290111 with (gcp + IPTG) or without (gcp) 100 µM IPTG. The error bars represent the standard errors or the means; n = 20. The star means the statistical difference; *p* < 0.05. The TEM is of the JW290011 *gcp*/*tsaD* conditional mutant JW290111 grown in TSB with (**D**) and without (**E**) 100 µM IPTG. The cell wall thickness is measured (**F**). The parental control JW290011 (CT) and the *gcp*/*tsaD* conditional mutant JW290111 with (*gcp* + IPTG) or without (*gcp*) 100 µM IPTG. The error bars represent the standard errors or the means; n = 20. The star means the statistical difference; *p* < 0.05.

Our previous studies demonstrated that Gcp/TsaD is essential for *Staphylococcus aureus* growth [4,15]. To further investigate its role, we utilized the previously constructed conditional *gcp*/*tsaD* mutant JW290111, in which the *gcp*/*tsaD* was placed under the control of IPTG-inducible P*spac* promoter. To tightly restrict the leaky activity of P*spac* promoter [32], a plasmid-encoded *lac I* repressor gene was provided to lower the basal transcription of *gcp*/*tsaD*. We examined the morphological changes in *S. aureus* following Gcp/TsaD depletion using scanning electron microscopy (SEM) and transmission electron microscopy (TEM). Compared to the cells with induced Gcp/TsaD expression (100 μM IPTG) (Figure 1A), the conditional mutant JW290111 exhibited a significant reduction in cell size (Figure 1B). A quantitative analysis of 20 randomly selected cells using iTEM software showed a 33.3% decrease in the average cell area, from 9.47 × 10^5^ nm^2^ in the wild type to 6.10 × 10^5^ nm^2^ in the mutant (Figure 1C). The TEM analysis revealed additional morphological changes: the Gcp/TsaD-depleted cells displayed a smoother surface and increased cell wall thickness compared to the induced cells, which maintained a rough and textured surface (Figure 1D–F), like their parental control strain [30]. Taken together, these results indicate that Gcp/TsaD plays a critical role in modulating cell morphology and cell wall biosynthesis.

### 3.2. Identification of Differentially Expressed Genes During Gcp/TsaD Downregulation

**Figure 2:**
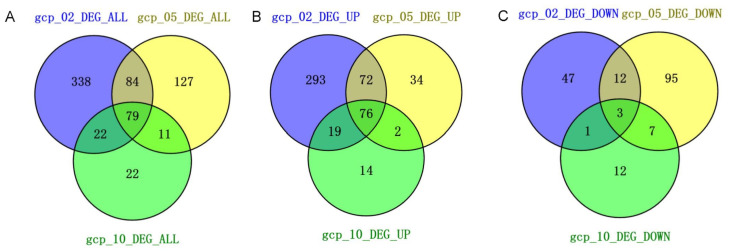
The diagram illustrates the overlapping differentially expressed genes at various growth phases during the downregulation of Gcp/TsaD. (**A**) The total differentially expressed genes for the IPTG-induced *gcp*/*tsaD* expression mutant JW290111 (gcp) in the absence and presence of 100 μM IPTG. (**B**) The differentially upregulated genes for the IPTG-induced *gcp*/*tsaD* expression mutant JW290111 (gcp) across the different growth phases in the absence and presence of 100 μM IPTG. (**C**) The differentially downregulated genes for the IPTG-induced *gcp*/*tsaD* expression mutant JW290111 (gcp) across the different growth phases in the absence and presence of 100 μM IPTG. gcp_02, gcp_05, and gcp_10 represent the growth of the IPTG-induced *gcp*/*tsaD* expression mutant in TSB without IPTG at OD600 ≈ 0.2, 0.5, and 1.0, respectively.

To elucidate the mechanisms underlying the role of Gcp/TsaD in growth and morphology, we performed RNA-seq analyses across the different growth phases (early log, mid-log, and early stationary) in the presence of 100 μM IPTG or absence of IPTG. The gene expression changes were assessed using DESeq, with thresholds of q-value ≤ 0.05 and log_2_ fold change ≥ 1. The IPTG addition in the control groups had a negligible impact on gene expression [30]. In contrast, the Gcp/TsaD depletion resulted in substantial transcriptomic changes, with 523, 301, and 134 differentially expressed genes (DEGs) identified at the early log, mid-log, and early stationary phases, respectively (Table 2 and Tables S1–S3; Figure 2A). Specifically, 460, 184, and 111 genes were upregulated, while 63, 117, and 23 genes were downregulated during depletion of Gcp/TsaD across the respective growth phases (Table 2 and Tables S1–S3; Figure 2B,C). Using VENN2.1, we identified 76 upregulated and 3 downregulated genes that were consistently differentially expressed across all three growth phases during Gcp/TsaD depletion (Figure 2B,C; Table S4). No overlapping DEGs were detected across the growth phases in the control group [30], indicating that Gcp/TsaD orchestrates a distinct and broad transcriptional program in *S. aureus*.

### 3.3. Gcp/TsaD Downregulation Alters the Transcription of tRNA Genes

Gcp/TsaD is essential for tRNA modification, particularly in the biosynthesis of threonylcarbamoyl adenosine (t^6^A) in tRNA [25,27,33,34]. Our previous studies established the critical role of Gcp/TsaD in t^6^A modification in *S. aureus* [15]. Consistent with these findings, our RNA-seq analysis revealed the significant impact of Gcp/TsaD depletion on the transcription of transfer RNAs (tRNAs) during the early log phase of bacterial growth. Specifically, a 2.5-fold reduction in Gcp/TsaD levels resulted in a marked decrease in the transcription of twelve tRNAs essential for protein synthesis, including tRNA-Pro, tRNA-Ile, tRNA-His, tRNA-Gly, tRNA-Lys, tRNA-Leu, tRNA-Gln, tRNA-Glu, tRNA-Arg, tRNA-Asp, tRNA-Trp, and tRNA-Tyr (Table S1). Interestingly, the transcription of tRNA-Ile and tRNA-Ala was induced during the mid-log phase under depletion conditions (Table S2), while no differentially expressed tRNA genes were identified in the early stationary phase (Table S3).

### 3.4. Gcp/TsaD Downregulation Induces Genes Involved in Cell Wall and Capsulae Biosynthesis

The RNA-seq analysis revealed that Gcp/TsaD depletion upregulated several genes associated with cell wall biosynthesis during the early log phase, including *dltA*, *dltB*, and *dltD* (Table S1), which are key genes in teichoic and lipoteichoic acid production [35]. Additionally, the transcripts for the *ssb* (single-strand DNA-binding protein) and genes in the *capABCDEFGLMN* operon, involved in capsule synthesis [36], were significantly increased from the early log through the early stationary phases (Tables S1–S3). The transcription of the *ilv-leu* operon (*ilvABCD*-*leuBCD*), essential for branched-chain amino acid biosynthesis [15], was also elevated. The qPCR confirmed the increased expressions of *capA*, *capG*, and *capP* in the Gcp/TsaD-depleted cells (Table 3), validating the RNA-seq findings.

### 3.5. Gcp/TsaD Depletion Reduces Virulence Gene Expression

The RNA-seq analysis unveiled the substantial downregulation of multiple virulence genes in the Gcp/TsaD-depleted cells. In the early log phase, transcripts of *spa* (protein A), *coa* (staphylocoagulase), *clfB* (MSCRAMM family adhesin clumping factor, ClfB), *sbi* (immunoglobulin-binding protein), *ecb* (complement convertase inhibitor), *sph* (sphingomyelin phosphodiesterase), *hlgA* (bi-component gamma-hemolysin HlgAB subunit), and serine proteases genes (*splB*, *splC*, *splD*, and *splF*, respectively), were significantly reduced (Table S1). The mid-log phase depletion further suppressed expression of *spa*, *sasD* (cell-wall-anchored protein), *coa*, *vwb* (von Willebrand factor-binding protein), *scpA* (cysteine protease staphopain A), *efb* (complement convertase inhibitor), *scb* (complement inhibitor SCIN-B), *ecb*, *sbi* (immunoglobulin-binding protein, Sbi), *CHIPS* (chemotaxis-inhibiting proteins), *lukH* (leukotoxin H), *hly* (alpha-hemolysin), *SSL11* (superantigen-like protein), and the two-component sensor *saeS*, a crucial virulence regulator [37] (Table S2). In the early stationary phase, virulence genes, including *lukG*, *lukH*, *sbi*, *hlgG*, *hlgC*, and *hyl*, were also downregulated (Table S3).

### 3.6. Gcp/TsaD Influences Multiple Metabolic and Regulatory Pathways

**Figure 3:**
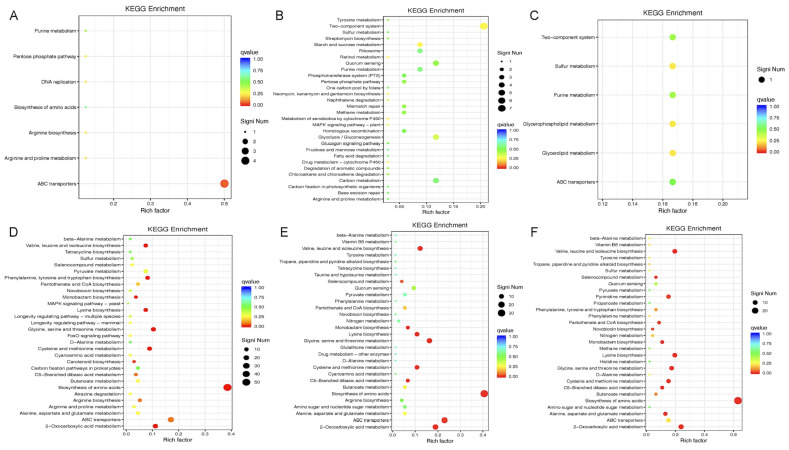
The significantly enriched pathways affected by downregulating Gcp/TsaD in *S. aureus* in a KEGG enrichment biological pathway analysis. (**A**) The significantly downregulated enrichment pathways caused by the depletion of Gcp/TsaD during the early log phase of growth (OD600 ≈ 0.2). (**B**) The significantly downregulated enrichment pathways caused by the depletion of Gcp/TsaD during the middle log phase of growth (OD600 ≈ 0.5). (**C**) The significantly downregulated enrichment pathways caused by the depletion of Gcp/TsaD during the early stationary phase of growth (OD600 ≈ 1.0). (**D**) The significantly upregulated enrichment pathways caused by the depletion of Gcp/TsaD during the early log phase of growth (OD600 ≈ 0.2). (**E**) The significantly upregulated enrichment pathways caused by the depletion of Gcp/TsaD during the middle log phase of growth (OD600 ≈ 0.5). (**F**) The significantly upregulated enrichment pathways caused by the depletion of Gcp/TsaD during the early stationary phase of growth (OD600 ≈ 1.0). The Rich Factor is the ratio of the differentially expressed number of genes in the pathway to the total number of genes in the pathway. The higher the Rich Factor, the higher the degree of enrichment. The QValue is the *p*-value after the multiple hypothesis test correction, and is in the range of 0 to 1; the closer the QValue is to zero, the more significant the enrichment.

**Figure 4:**
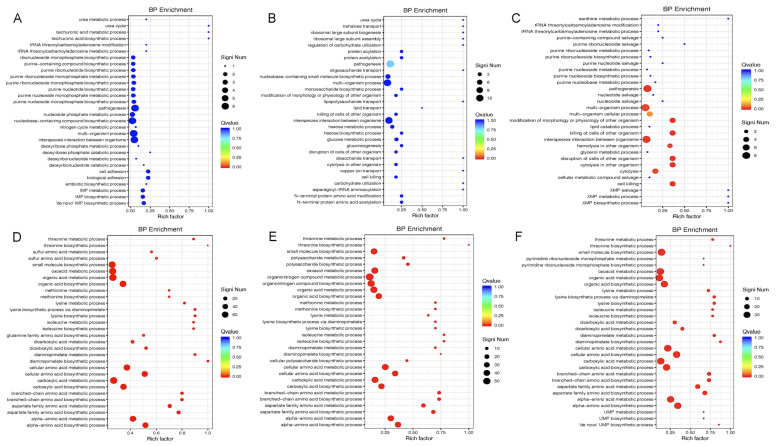
The significantly enriched pathways affected by downregulating Gcp/TsaD in *S. aureus* in a BP enrichment biological pathway analysis. (**A**) The significantly downregulated enrichment pathways caused by the depletion of Gcp/TsaD during the early log phase of growth (OD600 ≈ 0.2). (**B**) The significantly downregulated enrichment pathways caused by the depletion of Gcp/TsaD during the middle log phase of growth (OD600 ≈ 0.5). (**C**) The significantly downregulated enrichment pathways caused by the depletion of Gcp/TsaD during the early stationary phase of growth (OD600 ≈ 1.0). (**D**) The significantly upregulated enrichment pathways caused by the depletion of Gcp/TsaD during the early log phase of growth (OD600 ≈ 0.2). (**E**) The significantly upregulated enrichment pathways caused by the depletion of Gcp/TsaD during the middle log phase of growth (OD600 ≈ 0.5). (**F**) The significantly upregulated enrichment pathways caused by the depletion of Gcp/TsaD during the early stationary phase of growth (OD600 ≈ 1.0). The Rich Factor is the ratio of the differentially expressed number of genes in the pathway to the total number of genes in the pathway. The higher the Rich Factor, the higher the degree of enrichment. The QValue is the *p*-value after the multiple hypothesis test correction, and is in the range of 0 to 1; the closer the QValue is to zero, the more significant the enrichment.

To identify the affected biological pathways, we conducted KEGG and Gene Ontology (GO) enrichment analyses of differentially expressed genes. In the early log phase (OD_600nm_ ≈ 0.2), the downregulated genes were enriched in pathways including the ABC transporters, DNA replication, pentose phosphate pathway, and amino acid and purine metabolism (Figure 3A and Figure 4A). The GO terms included tRNA modification, nucleotide metabolism, pathogenesis, and cell adhesion. Conversely, the upregulated genes were associated with biosynthesis of amino acids (e.g., valine, leucine, and lysine), pantothenate and CoA biosynthesis, and multiple metabolic pathways (Figure 3D and Figure 4D). During the mid-log phase (OD_600nm_ ≈ 0.5), the downregulated genes were enriched in two-component systems, quorum sensing, ribosome biogenesis, DNA repair, and carbon metabolism (Figure 3B and Figure 4B). The upregulated genes again showed enrichment in amino acid biosynthesis and metabolism, as well as ABC transporters and quorum sensing pathways (Figure 3E and Figure 4E). In the early stationary phase (OD_600nm_ ≈ 1.0), the downregulated pathways included two-component systems, sulfur metabolism, purine metabolism, and glycerophospholipid biosynthesis (Figure 3C and Figure 4C), while the upregulated pathways included lysine, glycine, and methionine biosynthesis and ABC transporters (Figure 3F and Figure 4F).

**Figure 5:**
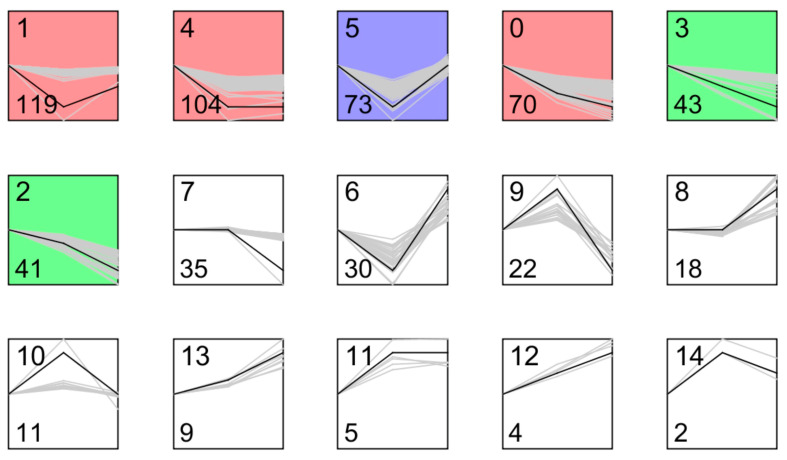
The interest trend class genes affected by Gcp/TsaD during the early log, middle log, and early stationary phases of growth in TSB. The profiles are ordered based on the number of genes assigned. The top number represents the profile number, and the bottom number represents the gene number in each panel. Different colors were used to distinguish the various classes of genes with distinct expression trend.

To further investigate the differentially expressed genes associated with Gcp/TsaD downregulation during various growth phases, we performed a trend analysis using the Short Time-series Expression Miner (STEM) [38] software (v1.3.13). The log fold changes (logFC) in the differentially expressed genes from the OD 0.2, 0.5, and 1.0 groups were used for this analysis, resulting in the identification of 15 gene expression trend clusters (profiles 0 to 14), as illustrated in Figure 5 and detailed in Table 4. With 167 genes in the class I trend of interest, the KEGG pathway enrichment analysis highlighted their involvement in lysine, carotenoid, and amino acid biosynthesis, as well as monobactam production (Table 5).

### 3.7. Identify Proteins That Potentially Interact with Gcp/TsaD in S. aureus

**Figure 6:**
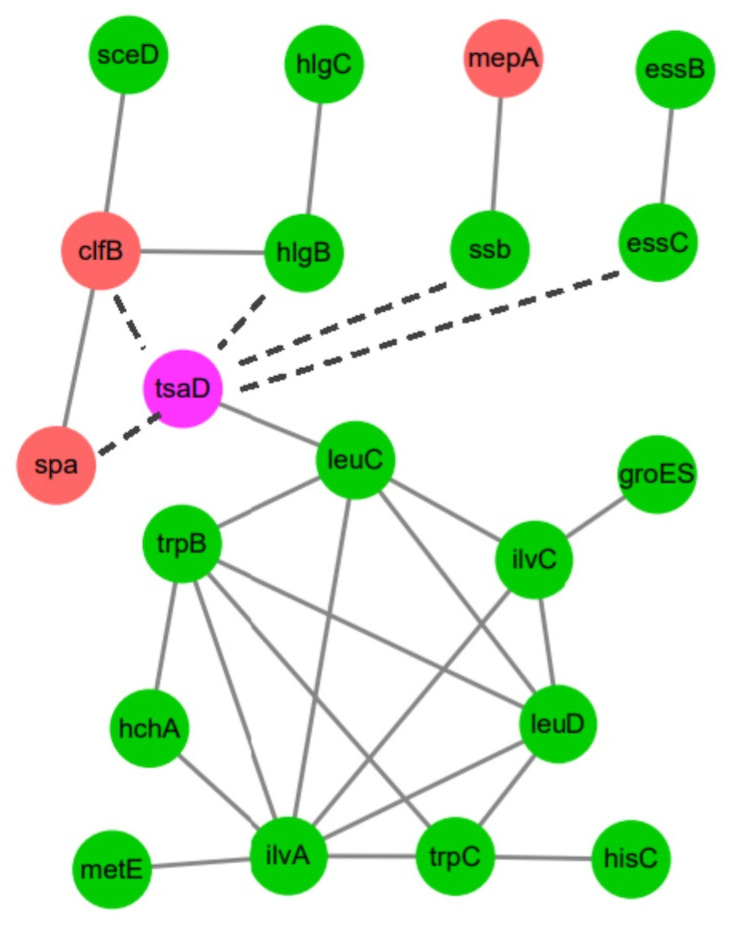
Protein–protein interaction network of the trend cluster I gene set. The red nodes represent genes with gradually increasing fold changes across the OD 0.2, 0.5, and 1.0 time points. The green nodes indicate genes with gradually decreasing fold changes across the same time points. The purple node represents the *gcp*/*tsaD* gene. Dotted and solid lines represent indirect and direct interactions, respectively.

To investigate the protein–protein interaction (PPI) landscape associated with Gcp/TsaD in *S. aureus*, we utilized the STRING v11.5 database, inputting the *gcp*/*tsaD* gene along with the 167 genes identified from the transcriptomic trend cluster of interest. Using a medium confidence interaction threshold (combined score ≥ 0.4), we identified a robust network of predicted protein interactions. Subsequently, we visualized the PPI network using Cytoscape version 3.6.1. The resulting network comprised 20 nodes and 24 edges (Figure 6), representing the genes and their predicted functional associations. The topological analysis revealed that several genes, such as *ilvA*, *trpB*, *leuC*, *leuD*, and *ilvC*, exhibited high connectivity degrees, suggesting their roles as potential regulatory or functional hubs.

Of particular interest, *leuC* (E5491_RS11545), encoding the large subunit of 3-isopropylmalate dehydratase (KO: K01703), demonstrated a direct interaction with the *gcp*/*tsaD* gene, indicating a potential central role in Gcp/TsaD-associated pathways. A functional enrichment based on the KEGG annotations linked *leuC* to multiple metabolism-related pathways, including valine, leucine, and isoleucine biosynthesis (ko00290); biosynthesis of amino acids (ko01230); C5-branched dibasic acid metabolism (ko00660); and 2-oxocarboxylic acid metabolism (ko01210). These interactions are detailed in Table 6.

### 3.8. Co-Expression Network Analysis of Genes Altered by Gcp/TsaD Depletion

To delineate the transcriptional network impacted by Gcp/TsaD, we constructed a co-expression network using the transcript abundance (TPM) data from the RNA-seq analysis of the *gcp*/*tsaD*-depleted mutant. The Pearson correlation coefficients between the gene expression profiles were calculated using the cor.test function in R. The genes with significant expression correlations (|r| > 0.7 and *p* < 0.05) with *gcp*/*tsaD* were defined as co-expressed and potentially co-regulated.

**Figure 7:**
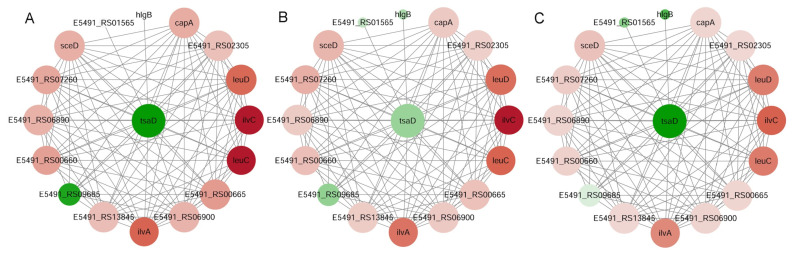
Dynamic regulatory network of the *gcp*/*tsaD* gene. The nodes represent the genes that follow the trend of interest from trend cluster I, and the node colors indicate the logFC values of gene expression differences between the uninduced and induced conditions. The edges represent significant correlations. Panel (**A**): OD 0.2; Panel (**B**): OD 0.5; Panel (**C**): OD 1.0.

This analysis identified 37 key genes that are significantly co-expressed with *gcp*/*tsaD***,** all of which were previously assigned to trend cluster I by our STEM clustering analysis. A dynamic regulatory network centered on *gcp*/*tsaD* was constructed and visualized using Cytoscape (Figure 7A–C). While the network architecture (nodes and edges) remained consistent across the growth stages (OD_600nm_ ≈ 0.2, 0.5, and 1.0), the node colors are differentiated to reflect the relative expression fold changes between the treated and untreated samples during each stage.

**Figure 8:**
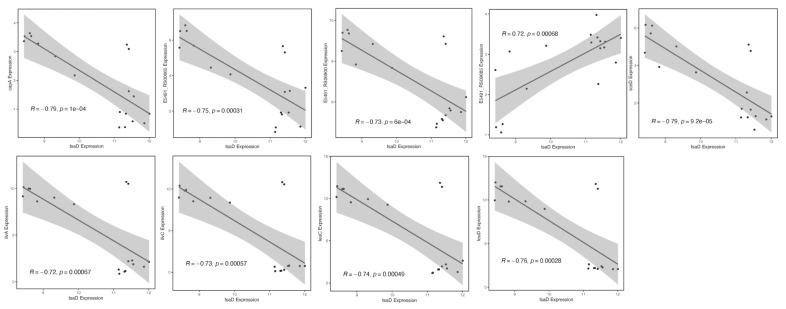
Correlation scatter plots between the *gcp* (*tsaD*) gene and key genes. The *x*-axis represents the expression values of the *gcp*/*tsaD* gene, while the *y*-axis represents the expression values of the corresponding key genes. Lines and gray areas highlight key genes that are closely correlated and similarly affected.

The topological analysis of this co-expression network (summarized in Table 7) identified additional key regulatory genes, including *hlg*, *sdpC*, *dhaK*, *idnK*, E5491_RS00880, *pruA*, and TPI. Correlation scatter plots depicting the gene expression relationships between *gcp*/*tsaD* and these targets were generated using the ggplot2 package in R (Figure 8), further supporting their tight co-regulation.

### 3.9. Gcp/TsaD Downregulation Increases Susceptibility to Fosfomycin, a Cell Wall Biosynthesis Inhibitor

Given the transcriptional and morphological changes associated with Gcp/TsaD depletion, we next evaluated its impact on bacterial susceptibility to various classes of antibiotics using standard MIC assays. While downregulation of Gcp/TsaD had no significant effect on the susceptibility to most of the antibiotics tested, it remarkably increased the susceptibility to fosfomycin by 16-fold (Table 8). Fosfomycin is a cell wall biosynthesis inhibitor that targets the MurA enzyme [39].

## 4. Discussion

Our study provides compelling evidence that Gcp/TsaD is a key contributor to multiple functions of *S. aureus* biology. Previous work from our group showed that Gcp/TsaD forms a complex with its operon partner YeaZ/TsaB, co-encoded in the *tsaBCDE* operon in *S. aureus* [28]. We further demonstrated that YeaZ/TsaB directly binds to the *ilv-leu* promoter to enhance its transcription [40]. Although Gcp/TsaD does not bind to the promoter, its depletion leads to a significant increase in *ilv-leu* transcription [15], suggesting that the Gcp-YeaZ complex may have co-functional roles in regulating ILV biosynthesis. Taken together, these observations suggest the possibility that Gcp/TsaD participates in broader regulatory circuits beyond its canonical role in tRNA threonyl-carbamoylation. Through the combination of a morphological analysis, transcriptomic profiling, network-based bioinformatic analysis, and antibiotic susceptibility assays, we demonstrated that Gcp/TsaD is involved in a wide array of physiological processes, including cell morphology, cell wall homeostasis, transcriptional regulation, virulence expression, and antibiotic response.

The transmission and scanning electron microscopy revealed striking morphological defects in the Gcp/TsaD-depleted strain, including reduced cell size and increased cell wall thickness. These findings indicate that Gcp/TsaD plays a crucial role in coordinating proper cell division and peptidoglycan biosynthesis. Our findings are consistent with previous reports that have shown the effects of depleted YgjD (Gcp’s homolog in *E. coli*) on bacterial cell morphology [41,42,43]. The increased cell wall thickness is likely a compensatory response to cell envelope stress and structural instability, a phenomenon that has also been observed in mutants defective in wall teichoic acid synthesis or penicillin-binding proteins [44,45]. Importantly, cell wall modifications are a common bacterial strategy to counteract environmental stresses, including host immune pressures and exposure to antibiotics [46]. The morphological phenotype in our Gcp/TsaD-depleted strain, therefore, likely reflects broader perturbations in the cell envelope integrity pathway. The changed cell wall structure might contribute to the increased resistance of autolysis and antibiotics-induced cell lysis in Gcp/TsaD-downregulated *S. aureus* [4].

The RNA-seq analysis revealed widespread changes in gene expression upon Gcp/TsaD depletion. The most dramatic alterations occurred during the early log phase, suggesting that Gcp/TsaD is particularly critical during rapid growth when the biosynthetic and translational demands are high. Notably, 79 genes were consistently differentially expressed across all three growth phases, indicating a core transcriptional response likely tied to Gcp/TsaD function. Among these, the genes involved in translation, energy production, and amino acid biosynthesis were prominently affected, consistent with Gcp/TsaD’s essential role in tRNA modification, specifically in the formation of the universally conserved threonylcarbamoyl adenosine (t^6^A) at position 37 of ANN-decoding tRNAs [25,33,47]. The t^6^A modification is essential for proper decoding during translation and for maintaining reading frame fidelity. In organisms ranging from bacteria to humans, loss of this modification results in translational stress, codon misreading, and ribosomal pausing [48,49,50]. The observed downregulation of multiple tRNA genes, including several involved in decoding ANN codons, provides further evidence that Gcp/TsaD depletion induces translational stress in *S. aureus*, thereby triggering global transcriptional reprogramming.

In response to this stress, we observed a compensatory upregulation of genes involved in cell envelope biosynthesis and modification. This included an increased expression of the *dltABCD* operon, which mediates D-alanylation of teichoic acids and contributes to resistance to cationic antimicrobial peptides [51], and upregulation of the *cap* operon, responsible for capsule synthesis. Capsules are known virulence factors that also play protective roles against host immune defenses [52,53]. These findings support the hypothesis that Gcp/TsaD depletion disrupts envelope homeostasis, resulting in the activation of envelope stress response pathways that aim to preserve cellular integrity under hostile conditions.

Interestingly, we observed marked downregulation of several key virulence genes, including *spa* (encoding protein A), *lukH* (Panton–Valentine leukocidin component), *scpA* (staphopain A), and *hlgA*/*B*/*C* (gamma-hemolysin subunits). These genes are under the control of major virulence regulators, such as the SaeRS two-component system and the Agr quorum-sensing network [37,54]. Our data indicate that Gcp/TsaD depletion leads to downregulation of *saeS*, suggesting that Gcp/TsaD may exert indirect control over virulence gene expression through modulation of regulatory circuits. Similar repression of virulence factors has been documented in *S. aureus* under oxidative stress or in response to translational arrest [55,56]. Thus, Gcp/TsaD appears to play a dual role in both maintaining basal metabolic functions and enabling the expression of pathogenic traits under favorable conditions.

The perturbation of metabolic genes upon Gcp/TsaD downregulation is also noteworthy. The pathways involving sulfur metabolism, purine biosynthesis, and branched-chain amino acid synthesis were broadly repressed, which is consistent with our previous findings [15]. These pathways are essential for nucleotide production, protein synthesis, and energy generation, especially under nutrient-limited conditions [57,58,59]. The network analysis further identified *leuC*, *ilvA*, and *trpB* as co-regulated hubs associated with Gcp/TsaD. These genes are key enzymes in leucine, isoleucine, and tryptophan biosynthesis, respectively, and have previously been linked to the virulence and persistence of *S. aureus* [60].

Importantly, our functional assays revealed that Gcp/TsaD depletion significantly increased susceptibility to fosfomycin, an antibiotic that targets the enzyme MurA, which catalyzes the first committed step in peptidoglycan biosynthesis [61,62]. This finding suggests that Gcp/TsaD may play a previously unappreciated role in maintaining cell envelope integrity or in the regulation of peptidoglycan precursor synthesis. The heightened susceptibility to fosfomycin supports a model in which Gcp/TsaD is functionally intertwined with cell wall biosynthetic pathways, potentially through direct or indirect regulation of enzymatic systems involved in precursor synthesis or membrane homeostasis. The precise mechanisms underlying these effects need to be further investigated. The hypersensitivity to fosfomycin likely reflects a compromised cell wall synthesis machinery and altered expression of cell wall biosynthetic genes. Given the increasing interest in adjuvant therapies that sensitize *S. aureus* to β-lactams and other cell wall-targeting antibiotics, our findings raise the possibility that targeting Gcp/TsaD or its downstream pathways could be a viable therapeutic strategy [63,64].

In conclusion, this study demonstrates that Gcp/TsaD plays a critical role in modulating global gene expression, morphogenesis, virulence, and antibiotic susceptibility in *S. aureus*. Its essential nature and broad regulatory influence highlight Gcp/TsaD as a promising target for novel antimicrobial development. Future studies will focus on elucidating the molecular mechanisms of Gcp/TsaD-dependent regulation and assessing its potential for therapeutic exploitation against drug-resistant *S. aureus* infections.

## Figures and Tables

**Table 1:** Primers for qPCR analysis.

Genes	Primers Name	Oligo Sequences (5′-3′)
*capA*	CapARTF435	TCCGAAGATTATGAGTGTGGATAAC
	CapARTR512	TGCACCGATTAGATTCACTACAG
*capG*	CapGRTF876	GAAGTTCCCTGGTGTCCTTATT
	CapGRTR959	CAACGGATTGGATTAGATTGTTATAGG
*capP*	CapPRTF864	GCTGACAGATTCTGGTGGTATT
	CapPRTR966	TGTGCCAATTACTCTCGATGTT
*ilvA*	IlvARTFor	AAGAGCACTCACACTTAATGCGCC
	IlvARTRev	GGCGTTGGTGCATCACATCAGTAT
*ilvD*	IlvDRTFor	CACCCGGTATGATTTAGCAG
	IlvDRTRev	ACAAGTAGGGCAGGCATTTTG
*leuA*	LeuARTFor	ACTTCTGCTTGGGCATCAGTACCT
	LeuARTRev	ACTTCTGCTTGGGCATCAGTACCT
*rrs*	16SrRNAFor-RT-JH	TCAGCGTCAGTTACAGACCA
	16SrRNARev-RT-JH	TAATACGACTCACTATAGGG

**Table 2:** Number of differentially expressed genes during Gcp/TsaD downregulation.

Group	IPTG−	IPTG+	DEG_Up	DEG_Down	Total
*gcp*/*tsaD* OD 0.2	gcp_02	gcp 02	460	63	523
*gcp*/*tsaD* OD 0.5	gcp_05	gcp 05	184	117	301
*gcp*/*tsaD* OD 1.0	gcp_10	gcp 10	111	23	134

*gcp*/*tsaD* inducible mutant strain JW290111.

**Table 3:** qPCR analysis of impact of Gcp/TsaD on transcriptions of genes of interest.

Genes	Fold Change (Increase) ^a^
*ilvD*	4.36 ± 0.47
*leuA*	79.36 ± 2.33
*ilvA*	124.24 ± 9.13
*capA*	16.56 ± 1.18
*capG*	5.66 ± 1.16
*capP*	17.39 ± 1.19

^a^ The fold change represents the transcription levels of genes with the depletion of Gcp/TsaD compared with those during the induction of *gcp*/*tsaD* transcription with IPTG (100 µM) at the exponential phase of growth (OD600 ≈ 0.5). The qPCR was repeated at least three times.

**Table 4:** The results of Short Time-series Expression Miner (STEM) analysis.

Profile	Count	Trend	Class
0	70	Down–down	Interest trend class I gene
2	41	Down–down	
3	43	Down–down	
12	4	Up–up	
13	9	Up–up	
9	22	Up–down	Interest trend class II genes
10	11	Up–down	
14	2	Up–down	
1	119	Down–up	
5	73	Down–up	
6	30	Down–up	
4	104	Down–flat	Interest trend class III genes
11	5	Up–flat	
8	18	Flat–up	Interest trend class IV genes
7	35	Flat–down	

**Table 5:** KEGG enrichment pathways of interest trend class I genes.

Group	Description	Count	*p*-Value
ko00300	Lysine biosynthesis	6	6.63 × 10^−5^
ko00261	Monobactam biosynthesis	4	0.000109
ko00260	Glycine, serine, and threonine metabolism	9	0.000111
ko01230	Biosynthesis of amino acids	18	0.000197
ko00270	Cysteine and methionine metabolism	7	0.000471
ko00290	Valine, leucine, and isoleucine biosynthesis	5	0.000824
ko01210	2-Oxocarboxylic acid metabolism	6	0.001309
ko00906	Carotenoid biosynthesis	2	0.043147

**Table 6:** The top 10 trend class I genes of interest involved in protein–protein interaction with Gcp/TsaD.

Genes Name	Gene Locus	Degree	Profile	Trend Type
*ilvA*	E5491_RS11555	7	2	down–down
*trpB*	E5491_RS07135	5	2	down–down
*leuC*	E5491_RS11545	5	2	down–down
*leuD*	E5491_RS11550	5	2	down–down
*ilvC*	E5491_RS11530	4	2	down–down
*trpC*	E5491_RS07125	4	0	down–down
*clfB*	E5491_RS14740	3	12	up–up
*hlgB*	E5491_RS13545	2	3	down–down
*hchA*	E5491_RS02895	2	3	down–down
*mepA*	E5491_RS01620	1	13	up–up
*gcp*/*tsaD*	E5491_RS11490	1		

**Table 7:** Degree analysis results of the *gcp*/*tsaD* dynamic regulatory network.

Gene	Gene Name	Degree	OD 0.2	OD 0.5	OD 1.0	Profile
E5491_RS11500	*tsaB*	37	−2.44891	−1.90448	−1.65545	
E5491_RS13535	*hlg*	26	−0.19841	−0.81054	−1.42237	3
E5491_RS13060	*sdpC*	25	0.131629	0.964253	1.408294	12
E5491_RS03405	*dhaK*	23	0.408297	−0.8035	−1.42473	0
E5491_RS14030	*idnK*	23	−0.94863	−2.19284	−2.63563	0
E5491_RS00880	E5491_RS00880	23	−0.50701	−1.21733	−1.63151	0
E5491_RS14300	*pruA*	22	−0.17791	−1.36565	−1.63798	0
E5491_RS04085	*TPI*	22	0.151122	−0.50351	−1.01814	3
E5491_RS00995	*ganQ*	21	−1.25819	−1.78034	−2.78774	2
E5491_RS01000	E5491_RS01000	21	0.18232	−1.41171	−2.5091	0
E5491_RS00985	*cycB*	21	−1.12371	−2.37355	−2.89513	0
E5491_RS14025	E5491_RS14025	21	−0.70145	−1.84441	−2.92043	3
E5491_RS00990	*ganP*	21	−1.21346	−2.40898	−3.11267	0
E5491_RS01005	E5491_RS01005	21	−0.72334	−1.43841	−2.45249	2
E5491_RS00745	*aldH*	21	0.105615	−0.73549	−1.22711	0
E5491_RS00980	*msmX*	21	−0.80904	−2.56146	−3.03886	0
E5491_RS15205	E5491_RS15205	20	−0.23762	−0.92482	−2.05323	2
E5491_RS11755	*yidC*	20	−0.04067	0.351541	1.042064	13
E5491_RS01510	E5491_RS01510	20	−0.28425	−1.18093	−1.54586	0
E5491_RS07475	*norB*	19	−0.66155	−1.96156	−2.48166	0

Note: In the title, OD 0.2 refers to the logFC value from the differential gene expression analysis between the uninduced and induced conditions at the OD 0.2 time point. Similarly, OD 0.5 and OD 1.0 also represent the logFC values at their respective time points.

**Table 8:** MIC of antibiotics against IPTG-induced *gcp*/*tsaD* conditional mutant.

Compound	JW290111	JW290111
IPTG (100 μM)	+	−
Novobiocin	0.03	0.03
Trimethoprim	4	2
Rifamycin	0.06	0.06
Chloramphenicol	16	16
Erythromycin	>64	>64
Kanamycin	64	64
Ampicillin	8	8
Penicillin-G	2	4
Bacitracin	>64	>64
Phosphomycin	64	4
Piperacillin	1	1
Vancomycin	0.5	0.5
Polymycin B	>64	>64
Triclosan	0.03	0.03

Note: All experiments were triplicated. MIC values are expressed in μg/mL.

## Data Availability

The original contributions presented in this study are included in the article and Supplementary Materials. Further inquiries can be directed to the corresponding author.

## Supplementary Materials

The following supporting information can be downloaded at: https://www.mdpi.com/article/10.3390/microorganisms13092111/s1, Table S1: Differentially expressed genes during Gcp/TsaD downregulation at OD 0.2; Table S2: Differentially expressed genes during Gcp/TsaD downregulation at OD 0.5; Table S3: Differentially expressed genes during Gcp/TsaD downregulation at OD 1.0; Table S4: Common differentially expressed genes across different growth phases following Gcp/TsaD depletion; Table S5: Whole-genome gene expression changes during Gcp/TsaD downregulation at various growth phases.
